# Iodine intake as a risk factor for thyroid cancer: a comprehensive review of animal and human studies

**DOI:** 10.1186/s13044-015-0020-8

**Published:** 2015-06-18

**Authors:** Michael B. Zimmermann, Valeria Galetti

**Affiliations:** Laboratory of Human Nutrition, Department of Health Sciences and Technology, ETH Zürich, Schmelzbergstrasse 7, LFV D21, CH-8092 Zürich, Switzerland; Laboratory of Human Nutrition, Department of Health Sciences and Technology, ETH Zürich, Schmelzbergstrasse 7, LFV E14, CH-8092 Zürich, Switzerland

**Keywords:** Iodine deficiency, Iodine excess, Iodine status, Iodized salt, Iodine supplement, Urinary iodine, Goiter, Nodule, Thyroid cancer

## Abstract

Thyroid cancer (TC) is the most common endocrine malignancy and in most countries, incidence rates are increasing. Although differences in population iodine intake are a determinant of benign thyroid disorders, the role of iodine intake in TC remains uncertain. We review the evidence linking iodine intake and TC from animal studies, ecological studies of iodine intake and differentiated and undifferentiated TC, iodine intake and mortality from TC and occult TC at autopsy, as well as the case–control and cohort studies of TC and intake of seafood and milk products. We perform a new meta-analysis of pooled measures of effect from case–control studies of total iodine intake and TC. Finally, we examine the post-Chernobyl studies linking iodine status and risk of TC after radiation exposure. The available evidence suggests iodine deficiency is a risk factor for TC, particularly for follicular TC and possibly, for anaplastic TC. This conclusion is based on: a) consistent data showing an increase in TC (mainly follicular) in iodine deficient animals; b) a plausible mechanism (chronic TSH stimulation induced by iodine deficiency); c) consistent data from before and after studies of iodine prophylaxis showing a decrease in follicular TC and anaplastic TC; d) the indirect association between changes in iodine intake and TC mortality in the decade from 2000 to 2010; e) the autopsy studies of occult TC showing higher microcarcinoma rates with lower iodine intakes; and f) the case control studies suggesting lower risk of TC with higher total iodine intakes.

**Electronic supplementary material:**

The online version of this article (doi:10.1186/s13044-015-0020-8) contains supplementary material, which is available to authorized users.

## Introduction

Thyroid cancer (TC) is the most common endocrine malignancy and in most countries, incidence rates have been steadily increasing over the past few decades, particularly in women. In 2012, the age standardized (world population) incidence rate was 6.1/100,000 women and 1.9/100,000 men [1]. Comparing populations around the world, there is a greater than ten-fold difference in incidence; high incidence areas (incidence rates greater than 10/100,000 women) are Japan and the Pacific Islands, Italy and several countries in the Americas. Incidence rates in developed countries are more than twofold higher than in developing countries (in women, 11.1/100,000 versus 4.7/100,000) [1]. If recent trends continue, thyroid cancer will be the fourth most common cancer in the U.S. by 2030 [2]. Papillary thyroid cancer (PTC) is by far the most prevalent subtype, in most countries accounting for greater than 80 % of thyroid cancer, but anaplastic thyroid cancer (ATC), because of its poor prognosis, accounts for a large portion of the mortality. Increased screening and diagnostic testing is likely the major, but may not be the only, contributor to the rising incidence of thyroid cancer [3–7]. Suspected risk factors include radiation exposure during childhood (whether from nuclear accidents, natural radiation or medical imaging) [8–10], obesity and the metabolic syndrome [11, 12], environmental pollutants [13, 14], a family history of thyroid cancer or thyroid disorders [15], and possibly, iodine intake.

It is clear that variations in population iodine intake are a primary determinant of benign thyroid disorders, such as goiter, nodules, and hyper- and hypothyroidism [16]. In contrast, the role of iodine intake in thyroid cancer remains uncertain, despite decades of study and debate. At the 1927 International Conference on Goiter, the eminent pathologist Carl Wegelin argued that thyroid cancer was more common in areas of endemic goiter, with frequency at autopsy varying from 1.04 per cent in central Switzerland, an endemic goiter region, to 0.09 per cent in Berlin, a non-endemic region [17]. He predicted a drop in incidence of thyroid cancer as endemic goiter disappeared due to iodized salt, with a lag time of 30 to 40 years. Iodized salt was introduced into a handful of countries in the 1920s and then increasingly since the 1960s; in 2015, around 100 countries have national iodized salt programs [18]. Although there has been a decrease in the prevalence of ATC in many of these countries, at the same time the incidence of PTC has increased, particularly since the 1980s, as discussed below. Because of this temporal pattern, several authors have suggested increasing iodine intakes are contributing to the increase in PTC, while others disagree.

The prevalence of goiter and thyroid nodules is higher in iodine deficient populations [19] and goiter and nodularity, in many cases, precede the development of thyroid cancer [20]. In animal studies, chronic thyroid-stimulating hormone (TSH) stimulation produces thyroid tumors, and because an increase in TSH is an adaptation to iodine deficiency, one might expect this could be the mechanism for an increased incidence of thyroid cancer in iodine deficient populations [21]. However, mean TSH may be actually lower in mildly iodine deficient populations than in populations with higher iodine intakes [16], and the highest incidence rates for thyroid cancer are in Japan, where iodine intake is high [22]. On the other hand, mortality from thyroid cancer tends to be higher in regions of endemic goiter because of more frequent advanced tumor stages at diagnosis and an increased ratio of more aggressive subtypes. Clearly, the links between iodine intake and thyroid cancer are complex, and are discussed in detail in this review.

Recommended daily iodine intakes from WHO/UNICEF/ICCIDD are 90 μg for infants and young children (0–59 months), 120 μg for children 6–12 years, 150 μg for adolescents and adults, and 250 μg for pregnant and lactating women [23]. To assess iodine intakes and status of populations, WHO recommend the use of spot urine collections to measure the urinary iodine concentration (UIC), expressed as the median in μg/L. National UIC surveys are often done in school-aged children because they are easy to reach through school-based surveys and their iodine status can be used as a proxy for the non-pregnant adult population [23]. The median UIC is an excellent biomarker of recent exposure to iodine in populations, because it reflects intake from all dietary sources [24]. The median UIC criteria for iodine nutrition are shown in Table 1 [23]. These WHO criteria are used throughout this review to describe iodine nutrition in populations as deficient, sufficient or excessive.

**Table 1:** Epidemiological criteria for assessment of iodine nutrition in populations based on median urinary iodine concentration [23, 24].

Median urinary iodine concentration (UIC)	Iodine intake	Iodine nutrition
<20 μg/L	Insufficient	Severe iodine deficiency
20–49 μg/L	Insufficient	Moderate iodine deficiency
50–99 μg/L	Insufficient	Mild iodine deficiency
100–299 μg/L	Adequate or more-than-adequate	Sufficient
≥300 μg/L	Excessive	Risk of adverse health consequences (iodine-induced hyperthyroidism, autoimmune thyroid disease)

## Review

### Animal studies

#### Thyroid cancer in animals fed iodine deficient or iodine excessive diets

In female rats, provision of iodine deficient diets for 6 to 20 months increases serum TSH and causes thyroid tumors in 54–100 % of animals, mainly follicular adenomas and follicular carcinomas [25–28]. Similar effects were observed in hamsters [29], where the malignancies reported were both follicular and papillary carcinomas. Correa and Welsh [30] fed rats (*n* = 30) for 9 months a diet containing excess iodine (≈120 mg of iodine per day) or a control diet. There was a 40 % increase in thyroid weight, and histologic changes included enlarged follicles with increased colloid lined by flattened epithelia, but no thyroid tumors were found.

#### Thyroid cancer in animals fed iodine deficient or iodine excessive diets and exposed to carcinogens

**Fig. 1:**
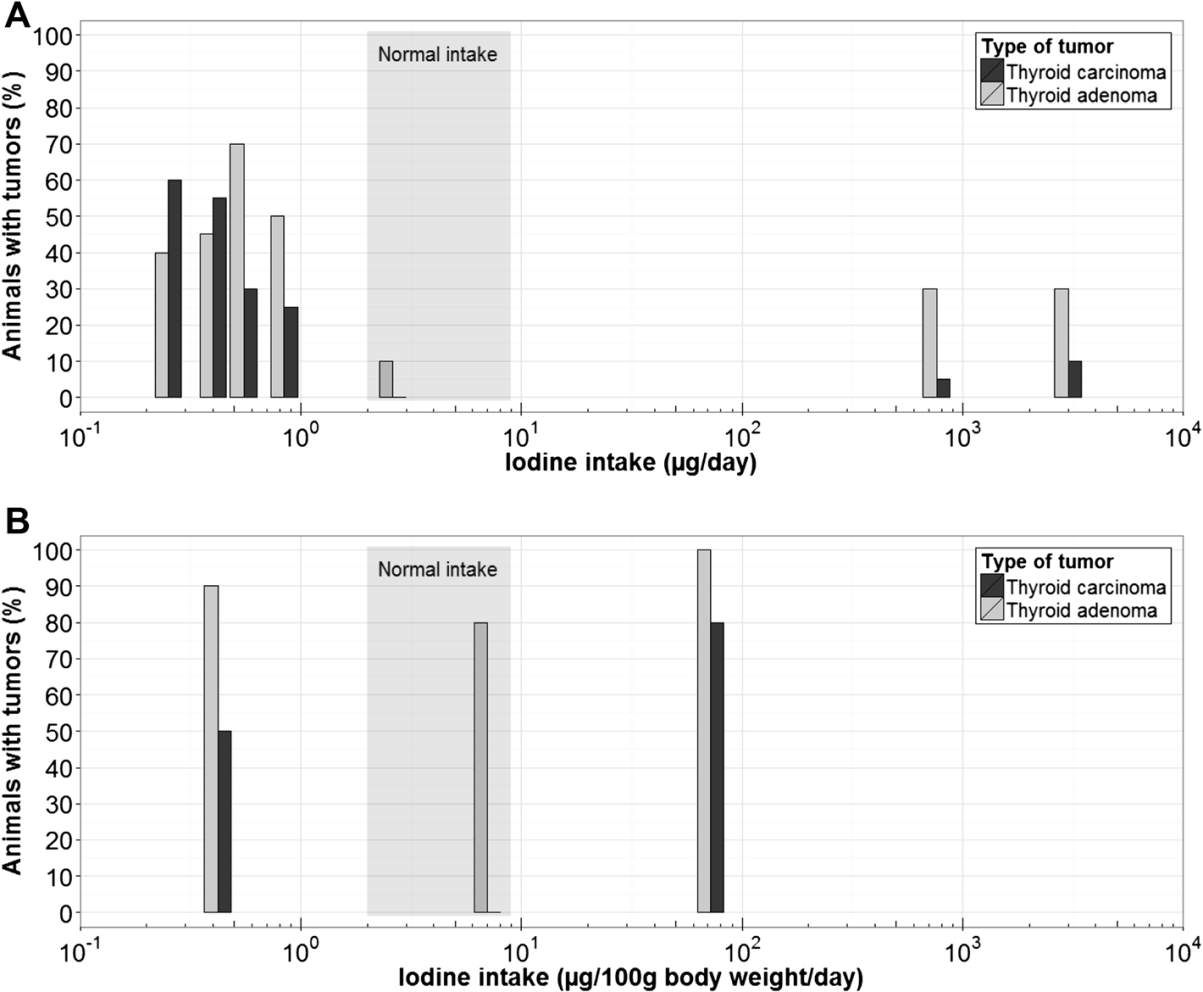
Prevalence of tumors in animals versus iodine intake. **a** Prevalence of animals with thyroid adenoma and thyroid carcinoma at week 26 after a single 2.8 mg/kg DHPN dose at week 2 and under one of seven long-term deficient, sufficient or excessive iodine diets (deficient intake: 0.25, 0.4, 0.55, 0.84 μg/day; normal intake: 2.6 μg/day; excessive intake: 760, 3000 μg/day) [33]. **b** Prevalence of animals with thyroid adenoma and thyroid carcinoma at week 110 after a single exposure to 4-Gy external radiation at week 6 and under one of three long-term deficient, sufficient or excessive iodine diets (deficient intake: 0.42 μg/100 g body weight/day; normal intake: 7 μg/100 g body weight/day; excessive intake: 72 μg/100 g body weight/day) [36]. Shaded area: range of normal iodine intake.

Thyroid tumor-promoting effects of iodine deficiency and excess have also been investigated in two-stage models in rats given carcinogens, such as N-bis(2-hydroxypropyI)-nitrosamine (DHPN) or N-nitrosomethylurea (NMU), and provided iodine deficient or excessive diets [31–33]. In iodine deficient rats, administration of NMU induces thyroid cancer after a shorter latency period and at higher incidence and multiplicity when compared to rats only iodine deficient or to rats that received NMU but were iodine sufficient [31, 32]. Kanno et al. [33] examined the potential thyroid tumor-promoting effects of iodine deficiency and excess for 26 weeks in a 2-stage model in rats given DHPN or saline. In saline-treated rats, iodine deficiency or excess alone was not carcinogenic, but in DHPN-treated rats, both iodine deficiency and excess increased thyroid follicular tumors, with iodine deficiency having a markedly stronger effect (Fig. 1a). In a similar 2-stage study that exposed rats to N-nitrosobis(2-hydroxypropyl)amine (BHP) and an excessive iodine diet [34], the incidence of thyroid cancer was 29 % in those fed the excessive iodine diet versus 33 % in those fed the iodine sufficient diet. To study the effects of iodine deficiency early in life on subsequent susceptibility to thyroid carcinogens, an iodine-free diet was fed to lactating rats and their weaned offspring until postnatal week 7, and then the offspring were exposed to DHPN [35], but this did not significantly increase thyroid tumors.

Boltze et al. [36] fed rats over a period of 110 weeks high (≈10 times normal), normal, and low (≈0.1 times normal) daily iodine intake and subjected them to single external radiation of 4 gray (Gy) or sham radiation. This study differed from the animal studies described above in that the induced iodine deficiency and excess were less severe and the experiments were done in younger rats. Iodine deficiency induced a doubling of serum TSH, while iodine excess had no effect on TSH. Alone, both iodine deficiency and excess increased the thyrocyte proliferation rate and induced thyroid adenomas, but induced no thyroid carcinomas. Combined with radiation, both iodine deficiency and iodine excess induced thyroid carcinomas (PTC and follicular thyroid cancer, FTC) in 50–80 % of animals, while iodine sufficient animals did not develop thyroid carcinomas (Fig. 1b). These data suggest both long-term iodine deficiency and excess are insufficient to stimulate thyroid carcinogenesis, but both promote thyroid carcinogenesis induced by radiation.

#### Conclusions

Overall, these animal studies suggest that iodine deficiency acts primarily as a promoter rather than as an initiator of thyroid carcinogenesis, or as a weak complete carcinogen. Iodine excess appears not to be an initiator, but may be a weak promoter. The relevance of the thyroid tumors produced in these animal experiments to human lesions is uncertain: most of the studies induced profound iodine deficiency and excess more severe than found in human diets, and, in general, the follicular tumors show a pattern of morphology and behavior different from human thyroid cancers.

### Proposed mechanisms linking iodine intake and thyroid cancer

#### Chronic TSH stimulation in iodine deficiency

Thyroid follicular cells proliferate only slowly under normal conditions, but in iodine deficient animals, serum TSH increases and the proliferation rate of thyroid cells increases by 5 to 30-fold [36], leading to marked thyroid hyperplasia and hypertrophy. Rapidly proliferating thyrocytes are likely more vulnerable to mutagens such as radiation, chemical carcinogens and oxidative stress, and may accumulate a higher number of genetic alterations. Thyroid hyperplasia induced by iodine deficiency results in chromosomal changes in the rat thyroid, with an increased number of aneuploid cells [37]. Several authors have suggested that thyroid tumors caused by iodine deficiency are due to chronic TSH overstimulation, possibly working together with epidermal growth factor and insulin-like growth factor I [38–41]. Consistent with this hypothesis, rat studies have shown that chronic TSH stimulation induced by goitrogen-containing diets or partial thyroidectomy result in a transition from thyroid follicular adenomas to follicular carcinomas [42–44]. This hypothesis is also consistent with the findings of increased FTC and ATC in populations with severe endemic goiter, because serum TSH is increased in moderate-to-severe iodine deficiency in an effort to maintain euthyroidism [16].

Excess iodide, by inhibiting thyroid hormone production (the Wolff-Chaikoff effect), can transiently increase TSH. However, in animal studies, chronic iodine excess does not increase serum TSH [36], and, in cell studies, moderate doses of iodide inhibit thyroid cell proliferation [45]. Most humans escape from the Wolff-Chaikoff effect as intrathyroidal iodide suppresses further iodide uptake via Na/I symporter inhibition [46]. Thus, the mechanism by which iodine excess promotes thyroid tumorigenesis is uncertain. Some population studies have reported slightly higher mean serum TSH in populations with sufficient or excess iodine intakes, compared to mildly deficient populations, but this is likely due to increased toxic adenomas in mild iodine deficiency [16].

#### Iodine, oxidative damage and apoptosis

Other mechanisms besides chronic TSH stimulation may contribute to thyroid tumorigenesis during iodine deficiency. Iodine deficiency increases H_2_O_2_-mediated, reactive oxygen species (ROS) generation, which can damage DNA and result in mutations [47]. Activity of antioxidant enzyme systems, including superoxide dismutase-3, are increased in iodine deficient rat thyroids, and this is accompanied by an increase of uracil and oxidized purine or pyrimidine adducts in thyroid DNA [48]. In immortalized thyroid cell cultures (TAD-2) and primary cultures of human thyroid cells, excess molecular iodide, generated by oxidation of iodine by endogenous peroxidases, induces apoptosis through a mechanism involving generation of free radicals [49]. Providing additional iodide to human thyroid cell lines reduces generation of H_2_O_2_ [50]. Exposure to iodine can also cause apoptosis in human thyroid cells and thyroid carcinoma cell lines through generation of iodolactones (iodinated derivatives of fatty acids) [51, 52].

#### Iodine intake and the BRAF mutation in PTC

Molecular alterations identified in PTC result in the activation of proteins along the mitogen-activated protein kinase (MAPK) pathway and include point mutations of the *BRAF* and *RAS* genes and *BRAF* and *RET* rearrangements [53]. *BRAF* mutations or rearrangements are found in 29–83 % of PTC, but are rare in FTC [54]. Several cross-sectional studies have looked at the effects of varying iodine status on the prevalence of the *BRAF* mutation in PTC. A study in southern Italy compared the percentage of PTC containing the *BRAF* mutation in two regions, one iodine sufficient and one iodine deficient; the individual iodine status of the cases was not defined [55]. The prevalence of *BRAF* positive PTCs was 107 out of 270 (39.6 %) in the iodine sufficient area and 18 out of 53 (33.9 %) in the iodine deficient area (*N.S.*). In China the prevalence of the *BRAF* mutation in PTC was significantly higher (69.2 %) in cases from a region with excessive iodine intake due to iodine rich drinking water (median UIC in the population was >900 μg/L) compared to 53.3 % in PTC cases from regions with mildly deficient to sufficient iodine intakes from iodized salt (median UICs of 82–198 μg/L) [56]. The overall incidence of PTC in the different regions was not reported. In contrast, in thyroid cells expressing activated *BRAF*, excess iodine may exert protective effects, attenuating acute *BRAF* oncogene-mediated microRNA deregulation [57].

#### Iodine intake and RAS mutations in thyroid cancer

Mutations in the *RAS* genes are present in 40–53 % of FTC and 6–51 % of ATC, but are rare in PTC [54]. A cross-sectional study compared the frequency of *RAS* oncogene mutations in thyroid tumors (25 adenomas, 16 FTC, and 22 PTC) from cases in an iodine sufficient area in Canada to cases in a mildly iodine deficient region in Hungary [58]. The *RAS* oncogene mutation rate was significantly higher in adenomas (85 versus 17 %) and FTC (50 versus 10 %), from the iodine deficient area than the iodine sufficient area, with no *RAS* mutations detected in PTC.

#### Iodine and RET rearrangements in thyroid cancer

*RET* rearrangements are reported in 13–43 % of PTC, but are rare in ATC and have not been reported in FTC [54]. Fiore et al. [59] reported that excess iodine may play a protective role during *RET/PTC3* oncogene activation in thyroid cells. They treated *RET/PTC3*-activated rat thyroid cells with 10^−3^ M sodium iodide, and found a reduction in cell proliferation and attenuation of the loss of Nis and Tshr genes and protein expression induced by *RET/PTC3* oncogene induction. When the human PTC cell line W3 and the FTC cell line FTC133 were incubated with excess iodine, progressively increasing iodine exposure first promoted (10^−3^ M iodine) and then inhibited (10^−2^ and 10^−1^ M iodine) growth and migration of thyroid cancer cells [60]. However, it should be remembered that the physiological concentration of iodine in the human thyroid is in the range of 10^−5^ to 10^−6^ M. Thus, thyrocyte exposure to iodine in these cell studies was several orders of magnitude above that encountered under physiological conditions resulting from varying dietary iodine intakes.

### Iodine intake and thyroid cancer: ecological studies

#### Sources of potential bias

Many ecological studies have examined the relationship between iodine intake and thyroid cancer incidence and mortality. The results of these studies should be interpreted with caution, because of multiple sources of potential bias [21]. It is difficult to compare thyroid cancer incidence across cancer registries because data collection methods are usually not standardized. The rate of thyroid microcarcinomas depends largely on the frequency and intensity of histological investigation of surgical and autopsy specimens; the higher the number of sections investigated per case, the more microcarcinomas can be found [61]. There is considerable observer variation in histological typing of thyroid cancer subtypes, particularly for FTC and mixed PTC-FTC, and this limits comparisons from different studies [62]. Comparing thyroid cancer rates across different time periods is biased by differences in histological classification, due to changes in 1974 and 1988 in the WHO classification system for thyroid cancer [63]; in 1988, WHO specified that all FTC presenting a papillary component should be considered PTC, and this likely contributed to the increase in the ratio of PTC:FTC in many countries after the classification change [64].

Because of its relatively low incidence, comparing rates of thyroid cancer between populations requires long periods of observation and this increases the likelihood of confounding from other risk factors that may have changed over the same time period. Also, there is likely to be a latency period after exposure of susceptible individuals to iodine excess or deficiency and subsequent changes in the thyroid cancer incidence. The length of this latency period is not known, with experts suggesting it could be 15 to 40 years [17, 65, 66]. There is also a long latency period in the elimination of goiter in endemic populations: for example, in central Switzerland, after three decades of salt iodization, the prevalence of goiter was <5 % in schoolchildren but was still 75 % in 50–60 year-olds [67]. An abrupt increase or decrease in population iodine intake might be expected to produce a period effect if all age groups were similarly affected, or a cohort effect if the effect were limited to a vulnerable age, such as childhood. Whether these effects confound longitudinal studies is uncertain.

It is particularly difficult to reliably compare thyroid cancer rates in populations with and without endemic goiter as a proxy for varying iodine intake. Ascertainment bias is high in these studies due to differences in the work-up of goiter and nodules, the frequency of surgical operations, and the indication for operation and preoperative diagnostic methods. The ratio of surgically removed goiters to non-operated goiters tends to be higher in non-endemic areas, because willingness of patients and physicians to perform operations is usually lower in endemic goiter areas. Also, because presence of a thyroid nodule has a higher likelihood of malignancy in iodine sufficient areas free of endemic goiter, there may be differences in diagnostic work-up [21].

Finally, in many countries, the introduction of iodized salt and an increase in iodine intake has coincided with the more widespread use of improved thyroid diagnostic tools (e.g., ultrasound, fine-needle biopsy and thyroid scintigraphy) and corresponding increases in incidence of PTC, often clinically silent. In France, an increase in the use of thyroid ultrasound in patients referred for evaluation of a thyroid disorder (from 3 to 85 %) and of cytology (from 4.5 to 23%) was associated with an increase in PTC incidence [68]. This introduces strong ascertainment bias in longitudinal or before and after studies favoring a higher incidence of PTC in more recent surveys. Thus, evidence from older longitudinal studies done before this increase in diagnostic intensity may be more valid than newer studies.

#### Iodine intake and differentiated thyroid cancer

**Fig. 2:**
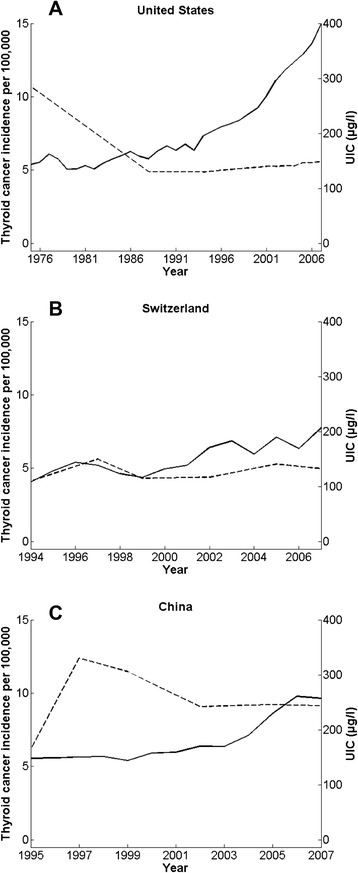
Thyroid cancer incidence and urinary iodine concentration evolution. National median urinary iodine concentration (UIC) (dashed line) as a proxy for iodine intake and age-standardized incidence rate of differentiated thyroid cancer (number of new cases per 100,000 per year) (solid line) in females of all ages in **a** United States, **b** Switzerland, and **c** China. Country data are from the Cancer Incidence in Five Continents (CI5) up to 2007 [69].

Differentiated thyroid cancer, comprising PTC, FTC and, rarely, Hürthle cell thyroid cancer, makes up about 95 % of all thyroid cancers. The following section discusses the possible links between changes in iodine intake and changes in rates of differentiated thyroid cancer in selected countries. In this section, Fig. 2a-c that plot country data on median UIC as a proxy for iodine intake versus incidence of thyroid cancer use comparable data from the Cancer Incidence in Five Continents (CI5) up to 2007 [69].

#### The U.S.

Based on the national UIC data from successive National Health and Nutrition Examination Surveys (NHANES I-III), the U.S. population had excessive iodine intake in the early 1970s (a median UIC of 320 μg/L), a more than 50 % decrease in iodine intakes to a median UIC of 145 μg/L in 1988 through 1994 [70], followed by sufficient and stable intakes during the period 2000–2010, with females having a median UIC of 142 μg/L and males a median UIC of 176 μg/L [71]. During this same period, incidence of thyroid cancer in the U.S. has been increasing by 6.6 % annually, and from 1973 to 2013, the annual incidence has increased by more than 500 % [72]. Thus, as shown in Fig. 2a, over past 4 decades in the U.S., while national iodine intakes have fallen from excess levels of intake to stabilize in the adequate range, there has been a steady increase in national incidence of differentiated thyroid cancer.

#### Northern Europe

Petersson et al. [73], using maps of goiter prevalence in Sweden from the 1930s to define iodine deficient versus iodine sufficient areas, found that during the period of 1958–1981, the relative risk of developing thyroid cancer was 0.92 in Swedish regions that were historically iodine deficient versus iodine sufficient. However, there was likely misclassification of exposure status as low-level iodine prophylaxis had been introduced in the formerly deficient areas before the study period. During the study period, the iodization level in salt was increased from 10 to 50 ppm and the iodine level in cattle feed supplements was increased, yet incidence rates of PTC increased similarly in both the iodine deficient and iodine sufficient regions, suggesting that the increasing rates were not related to original differences in iodine intakes.

Denmark introduced mandatory national fortification of salt used for bread making in 2000 and >90 % of Danish bread now contains iodized salt. This has improved iodine intakes but adults remain mildly iodine deficient with a median UIC of 83 μg/L because the fortification level is low, at 13 ppm [74]. The mean annual increase in the incidence of thyroid cancer in Denmark from 1943 to 2008 was 1.7 % in men and 1.8 % in women and was almost exclusively PTC [75]. The strongest increase in incidence began in the years before the iodization of salt, likely due to increased diagnostic activity. Because of differences in groundwater iodine content, before the introduction of iodized salt, the eastern part of Denmark was mildly iodine deficient (median UIC of 61 μg/L), while the western part was moderately deficient (median UIC of 45 μg/L). Although palpable goiter was found in 14.6 % in the area of moderate iodine deficiency versus 9.8 % in the area of mild deficiency [76], there were no regional differences in the overall incidence of thyroid cancer [77]. Also, the introduction of iodized salt did not result in a greater increase in the incidence of PTC in moderately iodine deficient western Denmark compared to eastern Denmark, suggesting that variations in iodine status are not a primary risk factor for thyroid cancer in the country [77].

In the Netherlands, the incidence of PTC has increased by only 2.1 % per year between 1989 and 2003, a less pronounced increase than in many other countries [78]. This might be partly explained by the stable and sufficient iodine intake of the Dutch population during the last 4 decades, together with the low level of radiation exposure and possibly the more conservative approach to incidentally discovered thyroid nodules [78].

The incidence of thyroid cancer is high in Iceland, a country with excess iodine intake from seafood and milk. In a study of surgical specimens for the period of 1944 to 1964 comparing Iceland and northern Scotland (where iodine intakes were presumed to be adequate), the PTC:FTC ratio was 6.5 in Iceland and 3.6 in Scotland [79]. The age-specific incidence rates for papillary carcinoma were approximately five times higher in Iceland than in Scotland in adults older than 35 years of age. It was hypothesized that high iodine intakes contribute to the high incidence of thyroid cancer in Iceland [79], but other authors have argued the high rates are due to the volcanic nature of the island [21]. Natural radiation is higher in volcanic areas, and radiation is known to increase risk for thyroid cancer, especially when the radiation occurs in childhood [21].

#### Central Europe

To correct severe endemic goiter and cretinism, iodized salt was introduced to northeastern Switzerland in 1923, and gradually spread to all Swiss cantons. In 1962, the iodine content in salt was increased from 5 to 10 ppm. A study analyzing more than 90’000 surgical specimens using the 1974 WHO classification reported a significant shift in the distribution of thyroid cancer subtypes comparing the period 1925 through 1941 to the period 1962 through 1973 [67]. In the former period, using age-adjusted data, 40 % of thyroid cancer was FTC, 38 % ATC and only 8 % was PTC. In the later period, 33 % were PTC, 30 % were FTC and 24 % were ATC. In a review of Swiss studies before 1973, the female:male ratio of thyroid cancer in areas of endemic goiter was 1.4 to 1.6, compared to those areas goiter-free or areas with iodized salt programs, where the female:male ratio was 2.1 to 3.0 [67]. In Geneva, between 1970–74 and 1995–98, when the iodine content and distribution of iodized salt did not change, the incidence of PTC increased from 0.7 to 1.8/100,000 for men and from 3.1 to 4.3/100,000 for women [64]. The authors suggested that the increasing incidence of PTC was mainly due to improved screening and diagnostic activity [64]. Figure 2b shows the relationship between Swiss national UIC data and national incidence of thyroid cancer for the period of 1994–2007; while iodine intakes in the population have remained stable, the prevalence of thyroid cancer has steadily increased.

In Germany, between 2003 and 2008, during which the country had sufficient iodine intakes from voluntary iodization of salt, the incidence rate of thyroid cancer rose from 2.7 to 3.4 (men) and from 6.5 to 8.9 (women) per 100,000 per year and was mainly PTC [80]. The incidence rate was higher in southern Germany, and the authors suggested this might be attributed in part to long-standing differences of iodine intake between different German regions, with the southern part of the country historically an area of iodine deficiency and endemic goiter.

Iodized salt was introduced into Austria in 1962 at a fortification level of 10 ppm. A study of thyroid cancer in surgical specimens in central Austria from 1952 to 1975 showed a significant increase in differentiated thyroid cancer and in the ratio of PTC:FTC, from 0.2 in 1952–1959 to 0.87 in 1970 to 1975 [66].

#### Southern Europe

Italy has one of the highest incidence rates for thyroid cancer; in 2007, the age-standardized (world population) incidence rate in women was nearly 20 per 100,000 women [1]. An Italian study analyzed thyroid cancer incidence from 25 cancer registries throughout the country between 1991 and 2005, before the national salt iodization program was introduced [81]. Populations located near the Alps and the Apennine Mountain Ranges, where iodine in soil and water is lowest, had low incidence rates for PTC compared to areas with higher iodine intake. However, the authors suggested this distribution of PTC was likely explained by local differences in medical surveillance, rather than iodine intake [81]. In a population-based study of ≈5600 subjects in Sicily in two regions with different iodine intakes during the period 1980 to 1990, surgery was performed in 792 patients on the basis of fine-needle biopsy of cold thyroid nodules. The frequency of thyroid cancer in patients with cold nodules was 5.3 % in the iodine sufficient area (mean UIC 114 μg/L) and 2.7 % in the iodine deficient area (mean UIC <50 μg/L) but was significantly different only in females [82].

A study in southern Greece examined patterns of thyroid cancer during the period from 1963 to 2000 [83]. The proportion of PTC was significantly higher in cases born in iodine sufficient areas (84 %, *n* = 162/193) than in cases born in previously iodine deficient areas (74 %, *n* = 159/214), while the iodine status of the current area of residence was not related to histological type [83].

In northwestern Spain, iodized salt was introduced in 1985. Rego-Iraeta et al. [84] studied rates of thyroid cancer during the period of 1978 to 2001 during the population’s transition from mild iodine deficiency to iodine sufficiency. Comparing the years before and after salt iodization, the PTC:FTC ratio increased from 2.3 to 11.5, and the thyroid cancer incidence increased in females from 1.56/100,000 in the period from 1978 to 1985 to 8.23/100,000 in period from 1994 to 2001, with most of the new cases being papillary microcarcinomas [84].

#### China

Several Chinese studies have pointed out the temporal association between the introduction of the national program of mandatory salt iodization in 1996 and a subsequent increase in incidence of PTC [85, 86]. As shown in Fig. 2c, during the period from 1997 to 2002, the national median UIC has fallen and then remained stable, while the national incidence of thyroid cancer has steadily increased. In Shanghai, from 1983 to 2007, the annual percentage change in thyroid cancer incidence in women was 4.9 % from 1983 to 2003 and 19.9 % afterward [85]. Teng et al. [87] did a five-year follow-up study of thyroid disorders in three regions of China, one with highly excessive iodine intakes from iodine-rich drinking water (median UIC of 635 μg/L), one with mildly excessive intakes from iodized salt (median UIC of 350 μg/L) and one that was borderline iodine deficient (median UIC of 97 μg/L). No significant differences among cohorts were found in the cumulative incidence of either single or multiple thyroid nodules, but the region with highly excessive intakes had 13 new cases of thyroid cancer while none were diagnosed in the other two regions [87]. In contrast, a large cross-sectional study found no correlation between iodine status and the prevalence of thyroid cancer in a coastal region of China [88]. A large cross-sectional study in Hangzhou in 2010 found a higher risk of thyroid nodules in adults consuming non-iodized salt versus iodized salt (odds ratio (OR): 1.36; 95 % CI: 1.01, 1.83) and a higher risk of thyroid nodules in those with low iodine intakes, but not excess iodine intakes [89]. There have been changes in other suspected risk factors for thyroid cancer in China over recent decades, including increasing exposure to industrial pollution [90] and increasing adiposity [91]. Moreover, rapid improvements in health care during the same period have led to increasing diagnostic intensity for thyroid cancer, and this bias likely explains a major portion of the increasing thyroid cancer incidence in China.

#### Australia

Burgess et al. [92, 93] using data from Tasmania’s and other regional cancer registries in Australia has reported regional and national thyroid cancer incidence and mortality trends, and related them to iodine status of the population. Tasmania and much of the Australian Eastern Seaboard were historically iodine deficient until about 1970, when a combination of iodization of bread (in Tasmania) and use of iodophors in the dairying industry improved iodine intakes. Iodized bread was discontinued in 1974 and the use of iodophors in dairying decreased in the early 1980s, resulting in the return of mild-to-moderate iodine deficiency in the 1980s and 1990s. In 1996, the median UIC in Tasmania had fallen to 42 μg/L, and in 2004, median UICs in the eastern states of Australia were 74 to 89 μg/L, indicating mild iodine deficiency [94]. During this period of falling and then deficient iodine intakes, for the period 1978–1998 in Tasmania [92] and for the rest of Australia from 1982–1997 [93], thyroid cancer incidence rates increased steadily. In the latter study, the increase was 6.7 % per year for females and 4.4 % per year for males, primarily due to a rise in PTC incidence. The average annual increase in PTC incidence was most marked in moderately-iodine deficient Tasmania (24.7 % per annum) and mildly iodine deficient Victoria (13.2 %), New South Wales (10.1 %) and Queensland (14.1 %), compared to the presumed iodine sufficient western states (4.0–8.9 %) [93].

#### Argentina

In a study from northern Argentina of the period from 1958 to 2007, where iodized salt was introduced in 1963, the incidence rate for thyroid cancer showed a progressive increase from 1.6 per 100,000 in 1960 to 3.6 per 100,000 in 2001, and the ratio of PTC:FTC increased from 1.7 to 3.9 [95].

#### Iodine intake and undifferentiated thyroid cancer

Although ATC is a rare form of thyroid cancer, accounting for less than 5 % of thyroid cancer in most countries, patients with ATC have a poor prognosis, and thus mortality from ATC accounts for most of the mortality from thyroid cancer [96]. In contrast to differentiated thyroid cancer, which often has a subtle clinical presentation and may be difficult to detect, ATC is correctly diagnosed in nearly all cases in countries with adequate health care because of rapid tumor growth and clinical presentation. Also, its highly specific histological features are easy to recognize [97]. Thus, compared to differentiated thyroid cancer, varying diagnostic intensity or criteria are much less likely to bias changes in ATC incidence rates within countries over time. For these reasons, ecological studies that describe changes in ATC incidence before and after introduction of iodized salt may be more reliable than for differentiated thyroid cancer.

Before and after studies of the effect of iodine prophylaxis on ATC have mainly been done in areas of historic endemic goiter in Europe. Bacher-Stier et al. [98] reported on changes in ATC in the Tyrol region of Austria. In the early 1960s, this area was moderately iodine deficient (mean UIC was 36 μg iodine/g creatinine) and goiter was endemic. Iodized salt fortified at level of 10 ppm was introduced in 1963 and at 20 ppm in 1992, and this resulted in iodine sufficiency and a mean UIC of 145 μg iodine/g creatinine by the mid-1990s. Overall incidence of thyroid cancer did not change from 1952 to 1995, but there was a marked shift in the percentage of thyroid cancer that was ATC, from 28.6 % in 1952–1976 to 4.9 % in 1986–1995 [98].

In Slovenia, edible salt was fortified with 10 ppm iodine from 1972 to 1997 and the population was mildly iodine deficient with a mean UIC of 83 μg iodine/g creatinine [97]. Salt iodization was increased to 25 ppm from 1998 to 2008 and this resulted in adequate iodine intakes and a mean UIC of 148 μg iodine/g creatinine. The annual incidence of ATC in Slovenia before 1998 was 3.25 per million versus 1.9 per million afterward [97]. From 1981 to 1995 in southern Germany, iodine status improved from moderate iodine deficiency (median UIC of 40 μg/L) in 1986 to mild iodine deficiency (median UIC of 72 μg/L) in 1997, and the percentage of thyroid cancer that was ATC decreased from 11.3 % to 7.3 % [99]. Other studies have described similar decreases in ATC after correction of iodine deficiency through iodized salt in Switzerland, Austria, Italy and Sweden [66, 67, 73, 100]. Along with improved medical care, the significant decrease in the incidence of ATC after the introduction of iodized salt in Switzerland may have contributed to the marked decrease in mortality from thyroid cancer between 1921 and 1978 [101].

However, it should be noted that other European countries have reported similar reductions in ATC without changes in population iodine intake. In Scotland, there has been a decrease in the incidence of ATC without changes in iodination policy [102]. Similarly in the Netherlands, where iodine intakes have been stable and sufficient over the past few decades, the incidence of ATC decreased by 7.1 % per year between 1989 and 2003 [78].

Northern Argentina in 1960 was severely iodine deficient and mean UI excretion was 20 μg/day [103]. After iodized salt was introduced, mean UIC increased into the sufficient range, and was 152 μg iodine/g creatinine in 1975. Over this period, the percentage of thyroid cancer that was ATC decreased from 15.2 % before salt iodization to 2.6 % after salt iodination, and the annual incidence decreased from 1.4 per million to 0.1 per million [103]. A plausible explanation for why the percentage of thyroid cancer that is ATC is higher in areas of severe iodine deficiency is that when goiter is endemic in a population, individuals are less concerned by the occurrence of thyroid nodules or swelling, and this may delay diagnosis in many cases until serious symptoms occur. This delay in recognition may allow initially well-differentiated thyroid cancer to change into anaplastic cancer [104, 105].

#### Conclusions

Table 2 summarizes the before and after studies looking at either the effect of the introduction of salt iodization or an increase in the salt iodization level on patterns of thyroid cancer. In three of eight studies that reported the gender ratio of thyroid cancer, the female: male ratio increased, while in two it decreased and in three it remained unchanged. The studies show a shift in the subtypes of thyroid cancer: all studies report an increase in the PTC:FTC ratio, and all but one show a decrease in the percentage of ATC. No studies have found an increase in incidence or frequency of ATC with increasing iodine intake. The findings in Table 2 are consistent with the earlier review of Williams [22], who reported that in countries with ‘high’ iodine intake (U.S., Iceland) the ratio of PTC:FTC ranged from 3.4 to 6.5, while in countries with ‘moderate’ iodine intake (the U.K. and northern Germany) the ratio was from 1.6 to 3.7, and in countries with ‘low’ iodide intake (Argentina, Columbia, Finland, southern Germany, Austria and Switzerland) the ratio was from 0.19 to 1.7.

**Table 2:** Before-and-after studies on the effect of salt iodization on type of thyroid cancer. The effect of introduction of salt iodization or an increase in the salt iodization level on the sex ratio (female: male, F:M) of affected subjects and the subtypes of thyroid cancer, by country: changes in the papillary thyroid cancer to follicular thyroid cancer ratio (PTC:FTC), and the percentage of anaplastic thyroid cancer (% ATC).

Country (reference)	Years pre-iodized salt			Year and change in salt iodization	Years post-iodized salt		
	PTC:FTC	% ATC	F:M		PTC:FTC	% ATC	F:M
Basel, Switzerland [148]	1944–1953			1962	1964–1973		
	0.27	28.7	2.5	Increase from 5 to 10 ppm	0.74	27.7	2.5
Zurich, Switzerland [67]	1925–1941			1962	1962–1973		
	0.19	36.9	1.3	Increase from 5 to 10 ppm	1.1	23.8	2.1
Innsbruck, Austria [66]	1952–1959			1963	1970–1975		
	0.21	na	0.9	Introduction at 10 ppm	1.1	na	1.9
Tyrol, Austria [98]	1952–1975			1963; 1992	1986–1995		
	0.55	28.4	na	Introduction at 10 ppm; increase to 20 ppm	1.5	4.9	na
Klagenfurt, Austria [149]	1984–1989			1992	1990–1995		
	2.6	na	na	Increase from 10 to 20 ppm	4.0	na	na
Krakow and Nowy Sacz, Poland [150]	1986			1997	2001		
	1.0	na	na	Introduction at 30 ppm	5.9	na	na
Lower Franconia, Germany [99]	1981–1985			1993	1991–1995		
	1.5	11.3	3.0	Increased use by the food industry at 20 ppm	3.4	7.3	2.2
Salta, Argentina [95]	1958–1972			1963; 1970	1985–2007		
	1.7	16.9	2.9	Introduction at 40 ppm; decrease to 33 ppm	3.9	6.4	4.0
Galicia, Spain [84]	1978–1985			1985	1994–2001		
	2.3	na	4.3	Introduction at 60 ppm	11.5	na	3.4
Parma, Italy [100]	1998–2003			2005	2004–2009		
	13.0	2.3	3.1	Introduction at 30 ppm	13.6	1.0	3.2
Shenyang, China [86]	1992–1996			1995; 2000	1997–2009		
	2.3	7.1	3.2	Introduction at 20–60 ppm; decrease to 35 ppm	21.9	2.1	3.6

Although the data in Table 2 suggest that populations in areas of sufficient iodine intake seem to have fewer of the more aggressive ATC and FTC, but more PTC, the comparisons may have been biased by other factors that may influence the presentation of thyroid cancer besides iodine intake. Interestingly, the increase in the PTC:FTC ratio and the decline in ATC is evident even in the study periods that preceded the introduction of increased diagnostic intensity for thyroid cancer. However, it should be remembered that increases in the incidence of differentiated thyroid cancer and the PTC:FTC ratio over the past two decades have occurred in countries with decreasing, stable and increasing iodine intakes. As shown in Fig. 2a-c, during the past several decades in the U.S., the incidence of thyroid cancer has been steadily increasing, but iodine intakes during the same period have decreased by about 50 %, and in Switzerland and China, two countries with well-established salt iodization programs, although iodine intakes have been stable, there have been steady increases in the incidence of thyroid cancer.

### Iodine intake and mortality from thyroid cancer

In most areas of the world, while the incidence of thyroid cancer has been increasing over the past few decades, mortality has steadily declined [1]. Decreases in thyroid cancer mortality are due mainly to improved early diagnosis and surgery or ^131^I therapy applied at an early tumor stage. However, because current or a history of goiter or thyroid nodules are strong risk factors for thyroid cancer [15], particularly the more aggressive subtypes (discussed below), it is possible that the substantial decline in iodine deficiency in many countries —particularly in low and middle-income areas that have introduced iodized salt— might also be contributing to the favorable trends in thyroid cancer mortality.

**Fig. 3:**
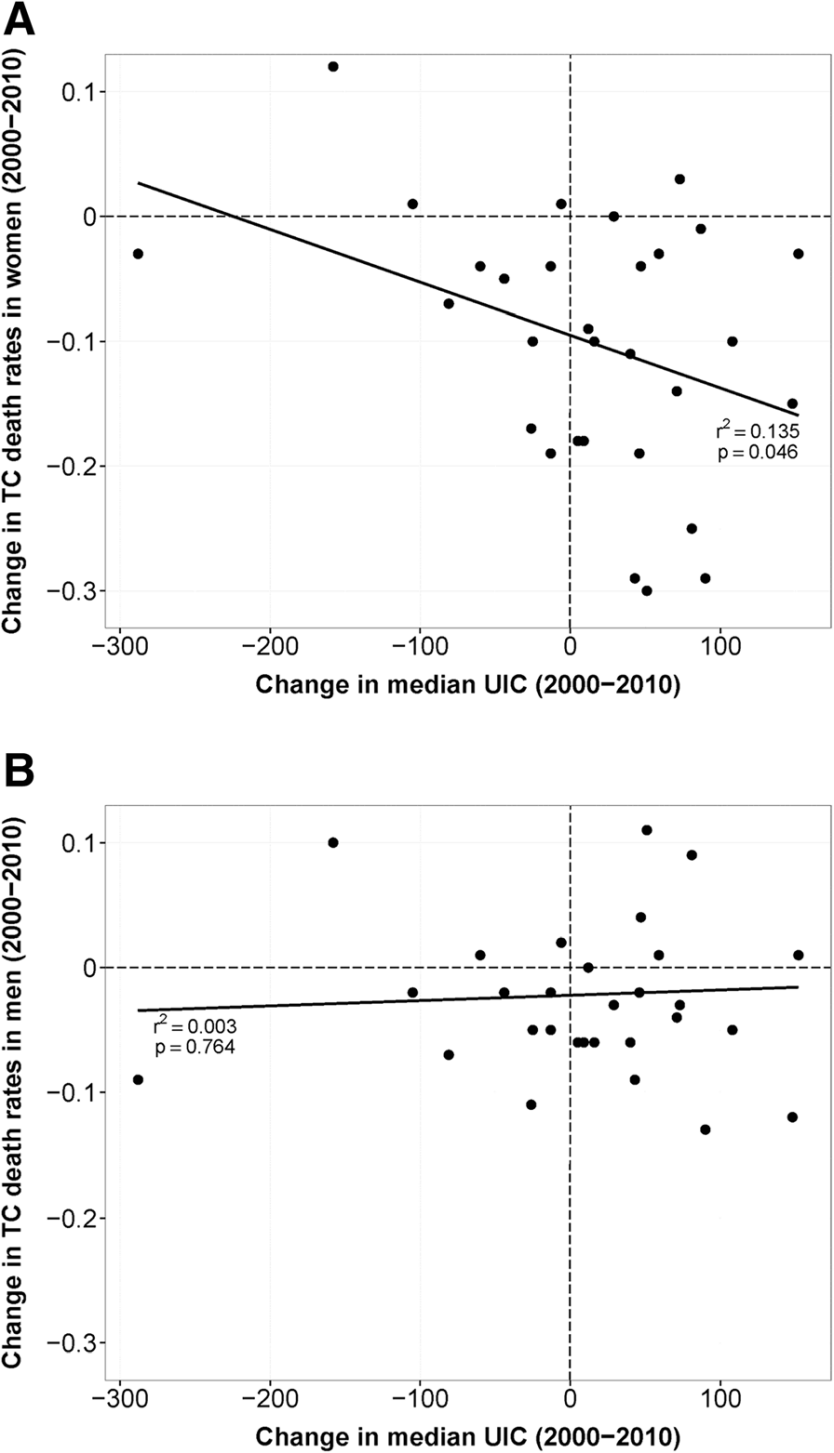
Change in thyroid cancer death rates versus change in median urinary iodine concentration. Correlation plot of change in age-adjusted (world population) death rates from thyroid cancer (TC) at all ages in **a** women and **b** men between 2000 and 2010 versus national or large subnational population median urinary iodine concentration (UIC) (μg/L) for the same time periods. Each data point represents one country. National thyroid cancer death rates are from La Vecchia et al. [1] and UIC data are from the Iodine Global Network [18] and WHO VMNIS [107]. Coefficient of determination r^2^ and p-value are derived from the linear regression model.

If population iodine status is a determinant of the pathogenesis of thyroid cancer, one might expect mortality rates in countries to be correlated with population iodine status. La Vecchia et al. [1], using data for thyroid cancer mortality and population size for countries in the period 1970–2012 from the WHO online database [106] estimated age-adjusted death rates from thyroid cancer at all ages in 2000 (1998–2002) and in 2010 (2008–2012) and percentage changes between these periods (Additional file 1: Table S1). Using these country data on thyroid cancer mortality, together with national or subnational median UIC data for same time periods, where available [18, 107], we calculated the change in age-adjusted death rates from thyroid cancer by gender between 2000 and 2010 versus the change in the population median UIC (μg/L) for the same time periods (Fig. 3). For women, there was a weak but significant indirect correlation (r^2^ = 0.135; *p* = 0.046) that was not present for men. These country data suggest that, for women only, a greater increase in iodine intake over the period 2000 to 2010 is associated with a greater decrease in thyroid cancer mortality.

### Iodine intake and occult thyroid carcinoma at autopsy

Occult thyroid carcinomas (OTC) are often incidentally found at autopsy, and nearly all are papillary microcarcinomas. Unlike clinical PTC, differences in the occurrence of OTC at autopsy are not influenced by differences in screening and diagnostic intensity, but more likely reflect true differences due to genetic and/or environmental factors [108]. Although differences in autopsy methods used for thyroid sectioning and histological examination may bias comparisons, associations between iodine intake in populations and the occurrence of OTC at autopsy may be relatively free of ascertainment bias and therefore valuable.

Kovacs et al. [109] compared rates of OTC at autopsy in two ethnically and socioeconomically comparable populations in Hungary, one in an iodine deficient area (median UIC of 70 μg iodine/g creatinine) and one in an area of iodine excess due to iodine-rich drinking water (median UIC of 500 μg iodine/g creatinine). Goiter prevalence was 22.5 % in the iodine deficient area versus 2.3 % in the iodine excess area, and thyroid nodularity was more common in the iodine deficient area. However, the occurrence of OTC in the two areas was not different: 4.95 and 4.52 %, respectively, and OTC were not more common in glands with nodular goiter, consistent with findings from an earlier Austrian study [110]. In contrast, in autopsy studies performed in Japan [111] and Finland [112], OTC was more common in glands with nodular goiter.

Table 3 shows the occurrence of OTC in autopsy series, categorized by population iodine status based on the median UIC at the time of the study. Because migration studies suggest that Japanese ethnicity is a strong determinant of risk for OTC [108], we analyzed the data in Table 3 without the five Japanese autopsy studies shown at the bottom of the table. We also excluded as an outlier a Finnish study that reported a prevalence of OTC of 35.6 % [112]. In the remaining studies, that examined mainly Caucasian and Hispanic populations, in areas with deficient (*n* = 11), sufficient (*n* = 10), and excessive iodine intake (*n* = 10), the weighted mean prevalence of OPTC was 5.3, 6.0 and 3.3 %, respectively. Comparing the weighted means, the prevalence of OTC was significantly lower (*p* < 0.01) in areas of excess iodine intake versus areas of sufficient and deficient intakes, but there was no significant difference comparing rates between areas of deficient and sufficient intakes. These data differ from two previous reviews that suggested higher iodine intakes were associated with an increase in prevalence of OTC at autopsy [22, 109]. However, the first review may have been affected by misclassification bias as iodine status was only broadly defined as high, moderate and low and criteria for this classification were not given [22]. The conclusions of the second review are limited in that nearly all of the iodine deficient populations included were Caucasian, while nearly all the iodine excessive populations were Japanese [109].

**Table 3:** Prevalence of occult thyroid cancer in adults at autopsy, by national iodine status. National population iodine status at the time of study as categorized according to WHO criteria for the median urinary iodine concentration (UIC) [23].^a^

Country (reference)	Year	No. autopsy cases	% with occult thyroid cancer
Probable deficient iodine intake (UIC < 100 μg/L) (*n* = 11)			
Italy [151]	1982	111	3.6
Chile [152]	1984	274	2.9
Poland [108]	1975	110	6.6
Portugal [153]	1979	600	1.0
Israel [154]	1981	260	4.2
Germany [155]	1987	1020	6.1
Spain [156]	1993	100	22.0
Belarus [157]	1993	215	8.8
Ukraine [158]	1996	162	10.8
Guatemala [159]	2005	150	2.0
Hungary [109]	2005	222	5.0
Mean			6.6
Weighted mean			5.3
Probable sufficient iodine intake (UIC = 100–299 μg/L) (*n* = 10)			
Canada [108]	1975	100	6.0
Sweden [160]	1981	500	6.4
USA [161]	1988	138	2.9
Brazil [162]	1989	300	1.0
Argentina [163]	1989	100	11.0
Iceland [164]	1992	199	6.0
Singapore [165]	1994	444	9.0
Austria [110]	2001	118	8.6
Greece [166]	2002	160	5.6
Turkey [167]	2011	108	3.7
Mean			6.0
Weighted mean			6.0
Probable excessive iodine intake (UIC ≥ 300 μg/L) (*n* = 10)			
USA [168]	1952	429	0.9
USA [169]	1955	1000	2.8
USA [170]	1955	221	1.4
USA [171]	1964	100	4.0
USA [172]	1966	300	2.7
USA [173]	1969	220	0.5
USA [174]	1974	157	5.7
Columbia [108]	1975	607	5.6
Hungary [109]	2005	221	4.5
Brazil [175]	2006	166	7.8
Mean			3.6
Weighted mean			3.3
Studies of Japanese populations in areas of excessive iodine intake (*n* = 5)			
USA, Japanese [176]	1971	100	24.0
Japan [174]	1974	1096	17.9
USA, Japanese [108]	1975	248	^24.2^
Japan [108]	1975	1167	28.4
Japan [111]	1990	408	15.7
Mean			22.0
Weighted mean			22.4

^a^Weighted means of iodine intake categories compared by using the Chi-squared test of independence: <100 μg/L vs. 100–299 μg/L (*p* = 0.244); ≥300 μg/L vs. <100 μg/L or 100–299 μg/L (both, *p* < 0.001)

### Goiter, benign thyroid nodules and thyroid cancer

Iodine deficiency sharply increases risk of nodules and goiter in populations [19]. In nodular goiter, distinct clusters of follicular cells are proliferating independent of TSH control [113]. Iodine deficiency may trigger formation of nodules through chronic stimulation by TSH and/or the mutagenic effects of increased reactive oxygen species in the iodine deficient thyroid [114]. Franceschi et al. [15] did a pooled analysis of case–control studies of benign nodules and goiter and thyroid cancer, including 2519 cases (2008 were PTC) and 4176 controls from 11 studies of goiter and 8 studies of benign nodules/adenomas. The studies of goiter included populations from five regions with sufficient to excessive iodine intakes (four studies from the U.S. in the early 1980s, and one from Japan) and six populations with sufficient to mildly deficient intakes (one from coastal China before the introduction of iodized salt and five from Europe). For women, ORs for a history of goiter for all thyroid cancer were 5.9 (95 % CI: 4.2, 8.1), and for PTC and FTC were 5.5 (3.9, 7.8) and 6.9 (3.8, 12.4), respectively. For men, the OR for all thyroid cancer was 38.3 (95 % CI: 5.0, 291.2). In that review, with the exception of a Japanese study where the OR for thyroid cancer was very high for women (26.5), the individual ORs from the other ten studies did not vary widely. For women, OR for a history of benign nodules/adenomas for all thyroid cancer were 29.9 (95 % CI: 14.5, 62.0), and for PTC and FTC were 28.9 (13.6, 61.2) and 62.3 (18.9, 205.8), respectively. The excess risk for goiter and benign nodules/adenomas was greatest within 4 years prior to thyroid cancer diagnosis, but a significantly elevated OR was still present more than 10 years before diagnosis. Case–control and cohort studies published since this analysis [15] have supported a link between goiter and/or thyroid nodules and risk of thyroid cancer [115–117].

Because iodine deficiency increases risk for goiter and thyroid nodules, these data suggest risk for thyroid cancer might be increased by iodine deficiency. However, these associations might also be explained by an increased likelihood of thyroid cancer detection in iodine deficient populations because of more frequent thyroid surgery. Also, there are other causes of goiter (e.g., thyroiditis) than iodine deficiency. In contrast to the results of the pooled analysis [15], studies in the U.S. [118] and Australia [119] did not find that thyroid cancer mortality was higher in historically goitrous regions in those two countries.

A meta-analysis of 14 cross-sectional or retrospective cohort studies [120] found the risk of thyroid cancer was significantly lower in multinodular goiter than in single nodules (OR 0.8; 95 % CI: 0.67, 0.96). However, there was moderate inconsistency across studies (I^2^ = 35 %): studies in iodine sufficient areas (U.S., Saudi Arabia, Nigeria, Croatia) found risk was lower in multinodular goiter than in single nodules (OR 0.77; 95 % CI: 0.65, 0.92), while studies in mildly iodine deficient areas (Italy, Turkey), where multinodular goiter would be expected to be more common, found no significant association (OR 0.88; 95 % CI: 0.68, 1.14).

### Total iodine intake and thyroid cancer

#### Hawaii, U.S.

A case–control study in Hawaiian adults reported the association between dietary iodine intake and thyroid cancer in 191 cases (85 % PTC) and 442 controls [121]. A food frequency questionnaire was used to estimate iodine intake, but iodine content of local seafood was not available, so values were taken from other sources. Geometric mean daily iodine intake (μg/day) was higher in female cases than in controls (394 versus 326, *p* = 0.01). Iodine containing supplements contributed ≈7 % of iodine intake. When the highest iodine intake quartile was compared to the lowest, there was a no significant increase in risk for thyroid cancer in women (OR 1.6; 95 % CI: 0.8, 3.2) or in men (OR 1.3; 95 % CI: 0.4, 3.7). Selection bias was not likely in this study as cases were selected from a population-based registry and controls selected through random sample of that population. However, ORs were adjusted only for age and ethnicity but not for other confounding factors.

#### California, U.S.

A case–control study of 608 women with thyroid cancer and 558 controls between the ages of 20 and 74 in northern California (51 % Caucasian and 35 % Asian) examined dietary iodine intake via a food frequency questionnaire and iodine levels measured in toenail clippings [115]. The main iodine sources were rice and pasta dishes and pizza (30 % of mean daily intake); milk and other dairy products (13 %); bread products (12 %); multivitamin pills (11 %); and fish/shellfish (3 %). A significant reduction in the risk of PTC was seen in the highest iodine intake quintile (>537 μg/day) compared to the lowest (<273 μg/day) (OR 0.49; 95 % CI: 0.29, 0.84), with a similar OR for Caucasian and Asian women. Iodine intake from food alone was not associated with risk, but iodine intake from supplements was; thus, the protective effect for total dietary iodine was largely attributable to the higher consumption of multivitamin pills (most brands contain 150 μg of iodine) by controls. Of note, the highest quintile of dietary iodine was greater than three times the WHO RNI for iodine for this age group. There was no association between toenail iodine and PTC, but the usefulness of nail clippings as an exposure biomarker is uncertain. Although this study had a large sample size and controlled for confounding by many risk factors, there is also the possibility of selection bias as cases were found from a cancer registry but controls were obtained through random-digit dialing.

#### New Caledonia

Truong et al. [122] performed a countrywide case–control study of iodine intake and thyroid cancer in women on the Pacific island nation of New Caledonia. The country has a high incidence of thyroid cancer but was not exposed to iodizing radiation from past nuclear testing in the Pacific. The study included 293 cases and 354 population controls. An extensive food-frequency questionnaire was used to estimate iodine intake over the previous 5-year period. Iodine intake was computed using a French food composition table, which did not have data for some local seafood, so iodine intakes may have been underestimated. Overall, total iodine intakes were low, and tertiles of intake were: <75.0, 75.0–112.6 and ≥112.6 μg/day, compared to the WHO RNI of 150 μg/day. There was no significant association of iodine intake and thyroid cancer: comparing the upper tertile of intake versus the lower tertile, the OR was 1.13 (95 % CI: 0.68, 1.87). A high consumption of cruciferous vegetables was associated with thyroid cancer among women with iodine intakes less than 96 μg/day (OR 1.86; 95 % CI: 1.01, 3.43).

#### French Polynesia

French Polynesia has one of the world’s highest incidence rates of thyroid cancer. A case–control study [123] included 229 cases of differentiated thyroid cancer (77 % PTC, 203 women, 26 men) matched with 371 controls. Daily dietary iodine intake was estimated from a food frequency questionnaire and was insufficient (<150 μg/day) in 60 % of both cases and controls. Dietary iodine intake (μg/day) was classified as: ≤74, severe or moderate deficiency; 75–149, mild deficiency, 150–299, optimal; and ≥300, excess. The ORs (95 % CI) for thyroid cancer at these intakes were: 2.57 (1.12, 5.93); 1.11 (0.63, 1.94); 1.00 (reference); 0.88 (0.38, 2.03) (p for trend = 0.04). These results did not change after adjustment with thyroid dose from prior exposure to radioactive fallout from nuclear testing in the area. A decreased risk of thyroid cancer was observed with a higher consumption of fish and shellfish, the main sources of iodine in the diet. More frequent iodized salt consumption was associated with an increased risk of thyroid cancer, but this result was likely biased because local doctors had advised patients who had a goiter or thyroid cancer to consume iodized salt.

#### Case–control studies: meta-analysis of total iodine intake and thyroid cancer

**Fig. 4:**
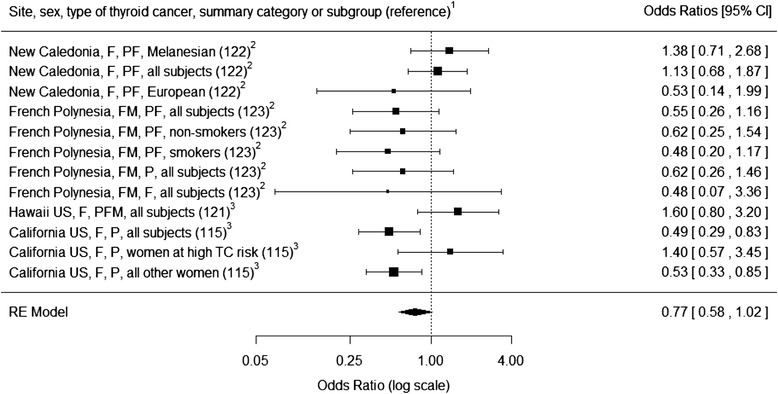
Forest plot for the association between thyroid cancer risk and daily iodine intake. Effect on thyroid cancer reported as adjusted odds ratios (OR) for the highest iodine intake quantile compared to the lowest quantile. Studies are ordered ascending from lower to higher iodine intakes. Overall pooled OR 0.77 (95 % CI: 0.58, 1.02; *p* = 0.068). Between-study heterogeneity: I^2^ = 42.6 % (*p* = 0.058). Publication bias: Kendall’s Tau = 0.061 (*p* = 0.841). ^1^Sex: female (F), male (M); type of thyroid cancer: papillary (P), follicular (F), medullary (M). ^2^Low daily iodine intake. ^3^High daily iodine intake.

We examined the association between iodine intake and thyroid cancer by performing a meta-analysis of pooled measures of effect available from the four case–control studies described above [115, 121–123]. We report the size of the effect on thyroid cancer as adjusted OR for the highest iodine intake quantile compared to the lowest quantile (Fig. 4). For each study’s subgroup, we algebraically derived the logarithmic estimate for OR (logOR) and its standard error (SE) from the available summary statistics. We calculated the overall pooled logOR and SE using random-effects model analysis with the DerSimonian and Laird method to estimate the between-study variance [124], and evaluated residual heterogeneity between studies using the I^2^ statistics. Publication bias was evaluated by visual inspection of the funnel plot of the random-effects model and by rank correlation test for funnel plot asymmetry. We performed data analysis with the R statistical programming environment (version 3.1.2) [125] using the metafor [126] and rmeta packages [127]. The meta-analysis indicates that the odds for thyroid cancer are 23 % less in the highest quantile of iodine intake versus the lowest, although this effect was only borderline significant (OR 0.77; 95 % CI: 0.58, 1.02; *p* = 0.068) (Fig. 4). There was moderate evidence for between-study heterogeneity (I^2^ = 42.6 %; *p* = 0.058), but evaluation of iodine median intake as potential source of heterogeneity in a meta-regression model revealed no evidence of linearity between iodine intake and thyroid cancer (*p* = 0.829). There was no strong evidence of publication bias (Kendall’s Tau = 0.061; *p* = 0.841).

#### Conclusions

In summary, two of the case–control studies (Hawaii and California) were done in areas of high iodine intakes, 2–3 fold higher than recommended intakes, while the other two were done in Pacific Island populations with mildly deficient intakes. The studies in California and French Polynesia suggest higher iodine intakes may be protective against thyroid cancer, while the other two show no association. Our meta-analysis of these studies suggests a trend toward lower risk for thyroid cancer with higher iodine intake. However, in these case–control studies recall bias is always possible as cases may recall diet information differently than controls. Also, food composition data on iodine in local foods was limited and was often derived from foods from other geographic areas. Finally, the main weakness of these studies is that they did not measure UIC to assess total dietary iodine exposure and did not have data on iodine intake from iodized salt added in the household, likely an important source dietary iodine. Thus, there is a high possibility of misclassification of case of iodine exposure.

### Case–control and cohort studies: seafood, milk and thyroid cancer

#### Seafood

Fish and shellfish are important sources of dietary iodine intake in populations that consume these products regularly. Liu and Lin [128] examined the relationship between fish consumption and risk of thyroid cancer in a meta-analysis of ten cohort or case–control studies. There was a 21 % decreased risk of thyroid cancer with high fish intake (OR 0.79; 95 % CI: 0.66, 0.94). Subgroup analysis was done comparing iodine sufficient populations in Norway and the U.S. to presumed iodine deficient populations in Italy, Sweden and French Caledonia. In the subgroup analysis, the summary OR was 0.74 (95 % CI: 0.59, 0.92) in the iodine deficient areas while no significant association was found in the iodine sufficient areas. Also, when the analysis was restricted to PTC, the authors found no significant association between fish intake and PTC risk. An earlier pooled analysis of case control studies published between 1980 and 1997 [129] from the United States, Japan, China and Europe found a borderline significant 12 % reduction in risk of thyroid cancer with high intakes of fish (OR 0.88: 95 % CI: 0.71, 1.1). There was a significant decrease in risk of thyroid cancer with high fish intake in regions with endemic goiter due to iodine deficiency (OR 0.65; 95 % CI: 0.48, 0.88) but not in iodine sufficient regions (OR 1.1; 95 % CI: 0.85, 1.5). The authors of these pooled analyses suggested that higher fish intakes exert a protective effect only in endemic goiter areas with suboptimal iodine intake.

#### Milk and milk products

In most European countries, the U.S. and Australia, population iodine intake from milk is typically greater than that from fish or seafood [130–132]. The native iodine content of milk is low, but the use of iodine-containing supplements for cows and iodophor disinfectants during dairying and transport result in high levels in milk and milk products [131]. Cross-sectional studies in iodine sufficient Chinese adults [89] and German university students [133] found no significant association between milk intake and the prevalence of thyroid nodules detected by ultrasound. A population-based case–control study in Sweden and Norway [134] reported a positive association of cheese (OR 1.5; 95 % CI: 1.0, 2.4) and butter (OR 1.6; 95 % CI: 1.1, 2.5) and thyroid cancer risk in adults, particularly in those who had lived in an endemic goiter area and had a high intake of all milk products. In a pooled analysis of four European case–control studies, high consumption of milk did not increase risk of thyroid cancer, but there was a significant increase in thyroid cancer risk for high intakes of cheese and butter (OR were 1.4 and 1.8 for the highest tertiles of intake) [135]. A later pooled analysis did not find a significant relationship of milk products and thyroid cancer [136] and case–control studies in iodine sufficient Poland [137] and New Caledonia [122] found no significant association between dairy consumption and risk of thyroid cancer. In a large U.S. cohort study of adults 50–71 years of age at baseline, during 7 years of follow-up there was no association between consumption of milk products (highest quintile had 1.4–1.6 dairy food servings per 1000 kcal) and risk of thyroid cancer in men (relative risk (RR) 0.78; 95 % CI: 0.45, 1.37) or women (RR 1.04; 95 % CI: 0.67, 1.62) [138].

### Iodine status and risk of thyroid cancer after radiation exposure

External radiation to the thyroid increases risk for thyroid cancer, particularly when the radiation occurs in childhood [9, 10, 139, 140]. The Chernobyl nuclear accident in 1986 exposed populations of Belarus, Ukraine, and the Russian Federation to internal radiation from radioactive iodines deposited in the thyroid, resulting in sharp increase in pediatric and adolescent thyroid cancer, mainly PTC [141]. Historically, the areas exposed to Chernobyl fallout were affected by varying degrees of iodine deficiency [142]. Chronic iodine deficiency increases thyroidal clearance of plasma iodine, increases thyroid blood flow and increases thyroid size, all of which may increase thyroid uptake of ingested radioiodines [142]. Also, iodine deficiency increases thyroid activity and thyrocyte proliferation, thereby increasing vulnerability of the thyroid to the accumulated radioiodine [54]. In rat studies, combined with a single external radiation dose of 4 Gy, both iodine deficiency and iodine excess induced thyroid carcinomas (PTC and FTC) in 50–80 % of animals, while iodine sufficient animals did not develop thyroid carcinomas (Fig. 1b) [36].

Thus, many experts predicted the risk of radiation-related thyroid cancer after Chernobyl would be higher in more iodine deficient areas, and a number of studies have tested this hypothesis [143–146]. However, at the time of the accident, there had been no recent assessment of iodine status of the populations in the most exposed areas, so it is difficult to know with certainty which of the exposed populations were more iodine deficient when the accident occurred. Therefore, a variety of surrogates have been used to categorize iodine status at the time of exposure, such as soil iodine concentrations [144], residence in a rural versus urban area [144] or measures of iodine status more a decade after the accident [143, 145, 146].

Shakhtarin et al. [143] investigated a population-based sample of 3070 individuals (2590 aged 6–18 years, and 480 adults) from 75 villages in the most highly contaminated regions of the Bryansk Oblast of Russia, a region that was historically an area of endemic goiter. UIC was measured in the participants in 1996 (a decade after the accident) and the median UIC was determined for each village and used to divide the study area into four zones, as follows: median UIC ≥100; 75–99; 50–74; or <50 μg/L. Thirty four cases of thyroid cancer were identified in those born in 1968–1986 who lived in the study area at the time of the accident in 1986 (20 in females and 14 in males, median age 16 years). The excess relative risk (ERR) of thyroid cancer was directly associated with increasing thyroid radiation dose and indirectly associated with median UIC. There was a significant combined effect of radiation exposure and iodine deficiency: at 1 Gy of exposure, the ERR in regions with a median UIC <50 μg/L (moderate iodine deficiency) was nearly two times that in areas of where the median UIC was ≥100 μg/L (iodine sufficiency).

Cardis et al. [144] performed a population-based case–control study of thyroid cancer in Belarus and Russia; cases were 276 thyroid cancers and controls were 1300 matched subjects below age 15 years at the time of the accident. Based on geologic maps of soil type in the affected areas, the authors estimated soil iodine concentration in rural settlements at the time of the Chernobyl accident and used these as a surrogate marker for iodine status, while classifying urban populations as iodine sufficient because it was assumed they were obtaining iodine sufficient foods from outside the local area. For subjects residing in areas with the lowest tertile of estimated soil iodine, the odds of developing thyroid cancer after a 1-Gy exposure were 3.2 (95 % CI: 1.9, 5.5) times higher than that for subjects living in areas of greater soil iodine. However, most of the areas studied had iodine deficient soils, so the comparison group (the higher two tertiles of soil iodine) was likely not iodine sufficient.

Tronko et al. [145] performed a prospective cohort study in 12 to 33 year-olds in the Ukraine who had been exposed to radiation from the Chernobyl accident during childhood. The authors assessed iodine status in subjects by goiter palpation and spot UICs more than a decade after the accident, in 1998–2000, when the median UIC was ≈50 μg/L. The study found no significant relationship between UIC and risk of thyroid cancer. A history of goiter was associated with a nonsignificant doubling in risk of thyroid cancer (OR 2.19; 95 % CI: 0.96, 5.03), but the study had limited statistical power due to a small number of cancer cases (*n* = 45). The later measurements of UIC and goiter may not reflect iodine status at the time of the accident because of changes in dietary habits and food distribution in the intervening period.

Zablotska et al. [146] screened nearly 12,000 individuals in Belarus aged 18 years or younger at the time of the Chernobyl accident. Past iodine deficiency was estimated by using self-reported history of diffuse and nodular goiter, as well as diffuse goiter and UICs measured during screening in 1996 through 2004. After adjustment for radiation dose, the prevalence rate for thyroid cancer was significantly higher for those with a self-reported history of nodular (OR 23.21) or diffuse goiter (OR 5.15), or with nodular or diffuse goiter diagnosed at screening (ORs 19.79 and 3.16). But UIC at screening was not associated with thyroid cancer; there was no significant increase in risk comparing those with moderate to severe iodine deficiency (UICs <50 μg/L) to those with higher UICs. However, Belarus began salt iodization in late 2000 and iodine status has generally been adequate in the population since then, so the later UIC measurements in this study likely differ from those at the time of the accident in 1986 [147].

#### Conclusions

In these studies, the reliability of the proxy measures used to define iodine status at the time of the accident is uncertain, and therefore it is difficult to draw conclusions. However, an error in classification of iodine status in these studies would likely have been random and would have biased their estimates toward the null. Despite this, 3 out of 4 studies [143, 144, 146] found that poorer iodine status in children at the time of the accident was associated with a ≈2–3 fold increase in risk for developing thyroid cancer.

## Conclusions

The incidence of thyroid cancer has been rising steadily over the past few decades in most countries [1]. Much of this increase is due to increased case finding because of improved screening and diagnosis methods, but there may also be a true increase, so it is important to clarify the role of suspected risk factors, including iodine intake. Population iodine intakes can vary up to 50 fold –from intakes less than 20 μg/d in areas of severe deficiency, to more than 1000 μg/d in areas with high iodine levels in drinking water or from intake of iodine-rich seaweed. Moreover, iodine intakes are shifting in many countries: they are increasing in some countries as iodized salt is introduced to correct iodine deficiency, while others report falling intakes, due to changes in dietary habits and patterns of iodine use by the food and dairy industry. It is well known that even small variations in population iodine intake are a determinant of benign thyroid disorders [16]. So why does the role of iodine intake in thyroid cancer remains uncertain, despite decades of study?

There are many reasons. Comparison studies of thyroid cancer epidemiology in populations and geographic areas are challenging because thyroid cancer is still a relatively rare and, in most cases, indolent cancer. Thus, long study periods in large populations are needed and this increases the likelihood of bias from changes in unmeasured risk factors other than iodine intake. There is the additional large uncertainty of the lag-time between changes in iodine exposure and changes in incidence of thyroid cancer; the lag-time between increasing iodine intake and the resolution of diffuse goiter and nodules in adult populations is several decades [67]. Accurate dietary assessment of iodine intake is notoriously difficult because discretionary use of household iodized salt and use of iodized salt in processed foods is difficult to quantify [24]. Finally, all of these difficulties are compounded by the current lack of an individual biomarker of iodine status: applying a population indicator such as the median UIC to classify individual risk for thyroid cancer likely introduces substantial classification bias.

Animal studies indicate iodine deficiency is a weak initiator but a strong promoter of thyroid cancer, mainly of the follicular type. Less convincing evidence suggests iodine excess may be a weak promoter of thyroid cancer. The proposed mechanism for the effects of iodine deficiency –chronic elevation of TSH stimulates thyrocyte proliferation and increases the likelihood of mutagenesis– is plausible and supported by animal studies. However, TSH is not elevated in animals fed diets with excessive iodine, so the mechanism for the effects of excess iodine is unknown. Limited data from thyroid cell cultures suggest exposure to high amounts of iodine reduces *RET/PTC3* and *BRAF* oncogene activation, but the relevance of these studies is uncertain because the iodine concentrations used were far above physiologic levels in the thyroid. A single human study found the *RAS* oncogene mutation rate was higher in FTC cases from an iodine deficient area, and in the two human studies investigating the *BRAF* mutation in PTC, one found no increased occurrence in cases from an iodine deficient area population while another found an increased occurrence in an iodine excess area. More well-controlled studies are needed to clarify the potential links between iodine intake and molecular alterations such as mutations of the *BRAF* and *RAS* genes and *BRAF* and *RET* rearrangements in thyroid cancer.

The overall incidence of thyroid cancer in populations does not appear to be influenced by the usual range of iodine intakes from dietary sources. The evidence is more convincing that increases in iodine intake may change the distribution of subtypes of thyroid cancer, particularly important are the data from countries before and after iodine prophylaxis done before the recent increases in diagnostic intensity (Table 3). These data are consistent in showing a higher percentage of the more-aggressive ATC and FTC in iodine deficient areas, whereas PTC seems to be more common in areas with high iodine intakes. Thus, by reducing FTC, and particularly ATC, iodized salt programs may be contributing to the decrease in thyroid cancer mortality seen in many countries; the data in Fig. 3 suggest this benefit may be strongest in women. However, firm conclusions cannot be made because of unmeasured covariates that may have changed over time along with iodine status, such as standards of medical care, environmental exposures, and histological classification.

Over the past 2 to 3 decades, there is clear temporal relationship in many countries between introduction of iodized salt and an increase in incidence of PTC. However, at the same time, several countries that have stable or decreasing iodine intakes, including Australia, the U.S. and Switzerland (Fig. 2), have also experienced an increase in PTC. Although a causal role of iodine intake in the etiology of PTC cannot be ruled out, a more likely explanation for the increasing incidence of PTC worldwide is the introduction and wider use of improved thyroid diagnostics. Compounding this, in areas without iodine deficiency and hence less goiter, individuals are more likely to notice a small change in their thyroid and go for medical examination. Thus, we may never have a clear answer to the question of whether changes in iodine intake contribute to PTC. Of note, autopsy studies of OTC are not confounded by changes in diagnostic and treatment, and they suggest that papillary microcarcinomas are actually less common in areas (outside of Japan) with high iodine intakes (Table 3).

The available data from case–control studies suggest higher iodine intakes and higher intakes of fish and seafood, particularly in iodine deficient populations, are linked to a small reduction in risk of thyroid cancer. However, the associations are weak and inconsistent and none of these studies were done in populations with clear iodine deficiency or excess. Future research should emphasize the collection of prospective data from large cohorts where total iodine exposure is assessed by measurement of repeated UICs, together with other iodine status biomarkers, such as serum thyroglobulin.

Overall, the available evidence suggests iodine deficiency is a risk factor for thyroid cancer, and that it particularly increases risk for FTC and possibly, ATC. This conclusion is based on: a) consistent data showing an increase in thyroid cancer (mainly follicular) in iodine deficient animals; b) a plausible mechanism (chronic TSH stimulation induced by iodine deficiency); c) consistent data from before and after studies of iodine prophylaxis showing a decrease in the percentage of thyroid cancer that is FTC and ATC; d) the indirect association between iodine intake and thyroid cancer mortality in the decade from 2000 to 2010; e) the autopsy studies of OTC showing microcarcinoma rates with lower iodine intakes; f) and the case control studies showing a trend toward lower risk for thyroid cancer with higher fish and total iodine intakes.

## Additional file

Additional file 1: Table S1. National age-adjusted death rates from thyroid cancer and median urinary iodine concentration in years 2000–2010.

## Abbreviations

ATC: Anaplastic thyroid cancer
BHP: N-nitrosobis(2-hydroxypropyl)amine
CI: Confidence intervals
TC: Thyroid cancer
DHPN: N-bis(2-hydroxypropyI)-nitrosamine
ERR: Excess relative risk
FTC: Follicular thyroid cancer
ICCIDD: International Council for the Control of Iodine Deficiency Disorders (now Iodine Global Network)
MAPK: mitogen-activated protein kinase
NHANES: National Health and Nutrition Examination Surveys
NMU: N-nitrosomethylurea
OR: Odds ratio
OTC: Occult thyroid carcinomas
PTC: Papillary thyroid cancer
RNI: Recommended nutrient intake
RR: Relative risk
SE: Standard error
TSH: Thyroid-stimulating hormone
UIC: Urinary iodine concentration
UNICEF: United Nations Children’s Fund
WHO: World Health Organization
