# Supplementary material for: Iodine intake as a risk factor for thyroid cancer: a comprehensive review of animal and human studies
Source: Thyroid Res. 2015 Jun 18;8:8. doi: 10.1186/s13044-015-0020-8 (PMC4490680; doi:10.1186/s13044-015-0020-8)
Supplement: Additional file 1: Table S1. — National age-adjusted death rates from thyroid cancer and median urinary iodine concentration in years 2000–2010. [file 13044_2015_20_MOESM1_ESM.pdf]

Supplemental Table 1. National age-adjusted death rates from thyroid cancer and median urinary iodine concentration in years 2000-2010.

Age-adjusted death rates from thyroid cancer are for women and men at all ages, by country. Year 2000 represents years 1998-2002 and year 2010 represents years 2008-2012. Population median urinary iodine concentrations (MUIC) (µg/L) are for the same time periods. National thyroid cancer death rates are from La Vecchia et al. [1] and UIC data are from the Iodine Global Network [18] and WHO VMNIS [107].

Country	Men, 2000	Women, 2000	MUIC, 2000	Men, 2010	Women, 2010	MUIC, 2010
Austria	0.52	0.61	101	0.39	0.32	191
Belgium	0.32	0.51	67	0.30	0.32	113
Bulgaria	0.36	0.47	111	0.32	0.33	182
Croatia	0.40	0.49	140	0.35	0.39	248
Czech Republic	0.42	0.61	120	0.33	0.32	163
Denmark	0.32	0.38	61	0.26	0.27	101
Finland	0.41	0.42	164	0.34	0.35	83
France	0.31	0.35	120	0.25	0.25	136
Germany	0.44	0.48	148	0.33	0.31	122
Hungary	0.55	0.57	80	0.43	0.42	228
Ireland	0.28	0.40	70	0.28	0.31	82
Italy	0.40	0.46	121	0.35	0.36	96
Latvia	0.40	0.89	59	0.51	0.59	110
Norway	0.34	0.45	117	0.29	0.26	104
Poland	0.33	0.56	103	0.27	0.38	112
Republic of Moldova	0.28	0.53	78	0.42	0.52	165
Romania	0.33	0.43	73	0.30	0.43	102
Spain	0.28	0.34	130	0.26	0.30	117
Switzerland	0.36	0.45	115	0.30	0.27	120
Argentina	0.25	0.35	180	0.23	0.30	136
Brazil	0.19	0.33	360	0.20	0.29	300
Chile	0.33	0.55	540	0.24	0.52	252
Costa Rica	0.15	0.57	233	0.24	0.32	314
Cuba	0.19	0.38	95	0.20	0.35	247
Ecuador	0.27	0.65	420	0.37	0.77	262
Mexico	0.32	0.62	176	0.33	0.59	235
United States	0.23	0.23	221	0.25	0.24	215
Venezuela	0.25	0.41	280	0.23	0.42	175
Australia	0.26	0.27	104	0.23	0.30	177
New Zealand	0.26	0.33	66	0.30	0.29	113
